# The Pioneer of Women Practitioners: Elizabeth Garrett Anderson, M.D. Paris, L.S.A.

**Published:** 1917-12-29

**Authors:** 


### THE PIONEER OF WOMEN PRACTITIONERS.

### ELIZABETH GARRETT ANDERSON, M.D. Paris, L.S.A.

Elizabeth Garrett Anderson has passed away in ripe old age, full of years and full of honours. She came of sturdy East Anglian stock, her father being Mr. Newson Garrett, of Aldeburgh, Suffolk, but she was born in London in 1836.

Mrs. Garrett Anderson was one of a large and very able family, one of her sisters, Mrs. Fawcett, being almost as well known as herself, and all her sisters being women of marked ability and strength of character. The family characteristics have passed on to the second generation, and show themselves not only in Mrs. Anderson's son and daughter, but also in her nephews, Sir Auckland and Sir Eric Geddes.

Miss Elizabeth Garrett was one of the three great pioneers of the women's branch of the medical profession. The other two early women practitioners in England were Dr. Elizabeth Blackwell and Dr. Sophia Jex Blake. The former was an M.D. of Geneva, U.S.A., and began to practise medicine before Miss Garrett obtained her qualification, but she was not on the British Register until 1858.

Miss Garrett's career as a student of medicine was beset with difficulties, and would probably have ended in failure had she been less firm of purpose and less patient in endeavour.

Part of her medical training was acquired by her acting as a probationary nurse at Middlesex Hospital, part by attending the ordinary science classes at that hospital, but most of her knowledge had to be gained by private coaching and by a five years' apprenticeship to a medical practitioner. Some work she put in at the London Hospital, some at St. Andrews University, and some at the extra-mural school at Edinburgh. It is difficult, if not impossible, for women students to-day to realise the amount of time, money, and strength that had to be expended on such a curriculum. That Miss Garrett carried it through to a successful end shows the wonderful tenacity and determination with which she was endowed.

The next necessity for qualification was to find an Examining Board who would accept her as a candidate for a degree or a diploma. The Universities and the Royal Colleges would not accept her, nor did the Society of Apothecaries desire that honour. However, they were bound to admit her by the terms of their Charter. They did so, and then banged, bolted, and barred their door. They admitted no more women for many years.

Five years later, in 1870, Miss Garrett took the degree of Doctor of Medicine of the University of Paris, and was warmly congratulated by Broca on her success.

Immediately after receiving the licence of the Apothecaries' Society—in 1866—Miss Garrett was appointed medical officer to the St. Mary's Dispensary in Wigmore Street. This institution was founded in order to enable poor women and children to enjoy the care of a woman doctor. It was simply a dispensary with which an outdoor maternity department was connected. This department was worked by midwives, but was under the responsible care of Miss Garrett, who was called by the midwives to all anxious and abnormal cases.

It is evident that the need for patient endeavour and untiring work did not cease for Miss Garrett when she qualified, nor even when she took the M.D. of Paris. She married in 1871, and added her husband's surname to her own. Mr. J. G. S. Anderson, of the Orient Line, became well known to his wife's colleagues and pupils, and owing to his geniality and strength of character he was a true yoke-fellow to his able, enthusiastic, but much burdened wife. Children blessed this union, but although she was a model wife and mother, Mrs. Garrett Anderson remained faithful to her resolve to open the doors of the medical profession to women, and her first care was to make this resolve operative.

Her dispensary grew and flourished, and in 1872 it developed into "The New Hospital for Women," which had its first home in two old houses in Marylebone Road. There was nothing splendid in this hospital except the work that was done in it, and the unconquerable spirit of its founder and chief medical officer. One does not know how else to describe Mrs. Garrett Anderson's function in the "Old New," as it was affectionately called. She was physician, surgeon, gynæcologist, and midwife all in one. She welcomed helpers and both recognised and awarded good work, but she reigned supreme—the unquestioned ruler over doctors, nurses, and patients alike. In fact it was the maternal despotism of Elizabeth Garrett Anderson that secured the continued existence—nay, more—the increasing prosperity of the infant institution. Among the more permanent of the fellow workers were Mrs. Garrett Anderson's sister-in-law, Mrs. Marshall, Dr. Louisa Atkins, and Mrs. de la Chervis. In 1888 another move was imperative. The leases of the two old houses in Marylebone Road expired, and new premises had to be secured as quickly as possible. Mrs. Garrett Anderson found a suitable site in the Euston Road, about midway between Euston and St. Pancras stations. Plans were drawn out and building operations were commenced. Meanwhile Mrs. Anderson and some medical women friends set to work to provide the necessary funds. Many meetings were held in all parts of London, in all the counties around London, and even further afield. Mrs. Garrett Anderson told the story of the beginning of the Women's Medical Movement in England, and demonstrated the need that existed for women surgeons and physicians. Mrs. Scharlieb followed her with a recital of the still more urgent needs of India, and Mrs. Marshall and other friends emphasised their statements. In a very little time a sum of approximately

**THE PIONEER OF WOMEN PRACTITIONERS**—(*continued*).

£30,000 was accumulated. The building rose as by magic under Mrs. Anderson's strenuous supervision, and in May 1899 Lady Salisbury opened the New Hospital at a very fine bazaar and fête, which both advertised its existence and assisted its finances.

It was proposed that the hospital should be named after her, or that it should be called the Elizabeth Hospital, but Mrs. Anderson's modesty and common sense finally convinced the building committee that it should retain its old name.

The New Hospital in Euston Road, even when first built and when it had only forty-two beds, was a well-found, well-equipped, and modern institution, in which good and scientific work could be done. The days of makeshift and the rabbit-warren premises were alike things of the past: the one bit of the old régime that survived was the vitalising and dominant personality of Mrs. Garrett Anderson.

The time, however, drew near in which, like other organisms, the New Hospital had to specialise its organs, and it became necessary to divide its medical and its surgical patients into separate wards under different officers. One of the evidences of Mrs. Anderson's statesmanship was the clearness with which she perceived that what anyone does constantly stands a fair chance of being well done, while the things that are done at long intervals can never become "secondary automatic." In obedience to this natural law Mrs. Anderson led the managing committee to appoint two surgeons, Mrs. Scharlieb and Mrs. Boyd, and two physicians, Dr. Julia Cook and Dr. Jane Walker. Dr. Ellaby was appointed ophthalmologist, but one great need was never supplied, and no children's ward was ever added to the New Hospital. The out-patient department was run by six women medical officers, who each attended two afternoons a week.

The success of the hospital was secured from the first. The beds were always full, and even when more and more were added the waiting-list of patients remained—and remains—deplorably long. The hospital has never wanted friends—at first for Mrs. Anderson's sake alone, later for her sake and for the sake of her colleagues, there were always wise and influential men and women to aid its councils, to direct its business, and to maintain its resources. Friendly observers and critics have visited its operating theatre and its wards, Sir James Paget, Sir Thomas Smith, Sir John Bland Sutton, Dr. Cullingworth, and Professor Gusserow among them. But "the old order changeth, giving place to new, and God fulfils Himself in many ways," so that at the present time neither Mrs. Anderson nor any one of her colleagues is on the staff of the New Hospital. She herself, Mrs. Boyd, and Dr. Cock have gone to their reward, and the others have passed on to other work. Still, the old spirit possesses the hospital, and the thoroughness, enthusiasm, and businesslike promptitude of its founder still informs and stabilises its work.

After the disastrous failure of the assault of the seven ladies on the Edinburgh University it was thought well to found a School of Medicine for Women in London. Among the prime movers in this enterprise were Mrs. Sophia Jex Blake, Dr. Chambers, Dr. Anstie, and Mr. A. T. Norton. The school was in working order in 1877, and all the necessary courses of lectures were provided, Mrs. Garrett Anderson being at first the only woman lecturer. Her subject was midwifery, and, subsequently, medicine.

The foundation of a school and the arrangement of a curriculum were essential, but of equal importance was the provision of hospital practice for the students and the securing of an open door into the profession through some body able and willing to grant degrees or diplomas. It was largely thanks to Mr. Garrett Anderson's "peaceful penetration" and to her business qualities that a reluctant consent was obtained from the Royal Free Hospital that the students of the school should be admitted to its clinical instruction. The Royal Free Hospital was the only one of the twelve great general hospitals in London that had not a male medical school attached to it. In the state of public opinion existing in the "'seventies" it was therefore the only hospital at all likely to permit the use of its clinical material to women students. The hospital was cold and reluctant—Mrs. Anderson and her friends were insistent and persistent. They had some money and more diplomacy, and with these combined forces they transformed the unfriendly neutral into a formal ally. There was, however, little or no *entente cordiale*, and it took many years and all Mrs. Anderson's astuteness and *savoir faire* to gradually infuse a gospel of love into the rigidly legal relations between hospital and school. In the long run Mrs. Anderson obtained a complete understanding, and in one of her happy speeches she compared the school to a well-dowered bride entering on an indissoluble relation with the hospital of her choice. And, she added, "the bride does not go empty-handed." The union has been deepened and perfected and now the school and the hospital are quite one—each an integral part of and essential to the other.

In the matter of qualification there was the difficulty that all the Universities, the Royal Colleges, and other qualifying bodies were legally unable to examine women and to grant them degrees or diplomas. Mrs. Anderson and many men who sympathised with her endeavours fought hard and yet patiently to overcome prejudice, and eventually they were successful. The Russell-Gurney Act, 1876, empowered all Examining Boards to admit women, and almost immediately the University of London and King's and Queen's Colleges of Physicians in Ireland opened their doors to women. The two Royal Colleges of Physicians and Surgeons in London were more obstinate, and did not yield until 1909. In consequence of the inability of women to gain these diplomas the great majority of the students of the School of Medicine for Women elected to take the degrees of the London University. They have been wonderfully successful in this endeavour, and the school founded by Mrs. Anderson and her friends claims a higher per-

**THE PIONEER OF WOMEN PRACTITIONERS**—(*continued*).

centage of University scholarships, medals, and honours than any other medical school in the Empire.

In the carrying out of Mrs. Anderson's great plan for the creation of a well-taught, well-endowed body of medical women, something more than professional education and graduation was essential. The women could never be the equals of and fellow-workers with the men unless they had equal professional-social advantages, unless they were amenable to the same internal professional discipline, and unless they learned and obeyed the same unwritten laws of professional duty, etiquette, and mutual consideration which are the pride and glory of our medical men. These laws and this *esprit de corps* are instilled into the juniors of the profession by personal contact with those elders who have for years observed and practised its best traditions. Opportunities for social intercourse, for informal teaching, and for discipline are chiefly afforded by membership of the great medical societies. In the days of Mrs. Anderson's great endeavour the most important body from this point of view was the British Medical Association. The British Medical Association, however, did not approve of women doctors, and refused to admit them. Mrs. Anderson herself obtained this privilege, but that was all. Efforts were made by her and by some liberal-minded men to obtain the desired boon, but it was not until 1892 that success was attained.

From this time on there has been a continued yielding of prejudice, and medical women are now eligible for the Fellowship of the Royal Society of Medicine and for all other important medical societies. The campaign has been won by Mrs. Garrett Anderson and her followers. A few minor posts still hold out, and ought to act as stimulants to the zeal of those women who are now leaders; such, for instance, as the opening of all medical appointments to men and women irrespective of sex, and determined only by the merits of the candidates.

Mrs. Garrett Anderson remained in the working world long enough to see the triumph of her endeavours and to enjoy the fruits of her labours. She saw her hospital come to vigorous maturity and to great prosperity. She saw the London Royal Free Hospital School of Medicine for Women become one of the recognised schools of the University of London. She saw nearly all the Universities and the two Royal Colleges conferring degrees and diplomas on the students of her school; and she saw her own daughter leading a hospital unit to France at the outbreak of the war. She saw the success, academic and professional, of many of the medical women who owed their training to her wisdom; and now that she has passed behind the veil to higher life and to clearer vision many are the women who rise up and call her blessed.

Mrs. Garrett Anderson had an unusual and most interesting personality. She was emphatically the right woman for the moment. She was endowed with just the qualities that were essential for her most difficult task, and with the perfect health without which her intellectual and moral gifts would have been sadly handicapped. Mrs. Anderson was a woman of one idea, and was able to subordinate every thing and every person to its realisation. She also had the necessary streak of hardness and grit. No difficulty daunted her; nor was she restrained from any action she thought desirable by any over-tender considerations. Entirely devoid of sentiment, she had a large fund of true kindliness, and while unable to understand the weakling's point of view, she never failed to give substantial and ready help to any case of real difficulty or genuine distress. Mrs. Anderson possessed an admirable sense of humour, which was very helpful to herself and to others in times of storm and stress. Some of her remarks deserve immortality, such as her reply to a young medical woman who grumbled that she had not sufficient scope. "Oh!" said Mrs. Anderson, "there is plenty of room at the top." To another who was, she thought, ambitious but inadequate, she advised, "People who wish to be leaders must lead."

And now Elizabeth Garrett Anderson has passed beyond our sight. The hospital, the school, and the medical women are her best memorial. To her may be addressed Shakespeare's lines—

Fear no more the heat of the sun,
Nor the furious winter rages,
Thou thy worldly task hast done?
Home art gone and ta'en thy wages.

Mary Scharlieb.

